# A retrospective multicenter study comparing the punctures to B2 and B3 in endoscopic ultrasound–guided hepaticogastrostomy

**DOI:** 10.1002/deo2.201

**Published:** 2023-01-03

**Authors:** Masanari Sekine, Yusuke Hashimoto, Taro Shibuki, Kei Okumura, Ikuhiro Kobori, Aki Miyagaki, Yoshihiro Sasaki, Yuichi Takano, Keita Matsumoto, Hirosato Mashima

**Affiliations:** ^1^ Department of Gastroenterology Jichi Medical University, Saitama Medical Center Saitama Japan; ^2^ Department of Hepatobiliary and Pancreatic Oncology National Cancer Center Hospital East Chiba Japan; ^3^ Department of Gastroenterology Dokkyo Medical University Saitama Medical Center Saitama Japan; ^4^ Department of Gastroenterology Toyooka Hospital Hyogo Japan; ^5^ Department of Gastroenterology National Organization Disaster Medical Center Tokyo Japan; ^6^ Department of Gastroenterology, Fujigaoka Hospital Showa University Kanagawa Japan

**Keywords:** bile duct of segment 2, bile duct of segment 3, endoscopic ultrasonography, endoscopic ultrasound–guided biliary drainage, EUS‐guided hepaticogastrostomy

## Abstract

**Objectives:**

In recent years, endoscopic ultrasound–guided hepaticogastrostomy (EUS–HGS) has been performed as an important salvage option for failed endoscopic retrograde cholangiopancreatography for biliary drainage. However, technical issues, such as puncture site (bile duct of segment 3 [B3] or bile duct of segment 2 [B2]), dilation method, stent selection, and procedural safety, need to be resolved for the optimization of EUS–HGS. The present study was to compare the safety, difficulty, and technical and functional success between biliary access via B2 and B3 during EUS–HGS.

**Methods:**

We conducted a retrospective investigation of 161 consecutive EUS–HGS cases across a total of 6 facilities, including those at our hospital. The patients were divided into two groups according to the successful drainage route: the puncture to B2 (P‐B2) or the puncture to B3 (P‐B3). We compared the technical and functional success rates, technical difficulty, and adverse events between the two groups. We also conducted a subgroup analysis to show the factors related to the procedure time.

**Results:**

There were 92 cases in the P‐B2 group and 69 cases in the P‐B3 group. There were no significant differences in the technical success, functional success, or adverse events between the groups; however, the procedure time was significantly shorter in P‐B2 cases than in P‐B3 cases. The multivariate analysis showed that the puncture site was the only factor related to the procedure time.

**Conclusions:**

Based on these findings, P‐B2 appears useful and safe. P‐B2 is as effective as P‐B3 and was able to be performed in a shorter period of time. The B2 approach can be considered a useful option for EUS–HGS.

## INTRODUCTION

Endoscopic ultrasound–guided biliary drainage (EUS–BD) was initially reported by Giovannini *et al.* (2001) as an EUS–guided technique for bilioduodenal anastomosis.^1^ Of these transmural procedures, EUS‐guided hepaticogastrostomy (EUS–HGS), which involves transgastric and hepatic drainages, was first reported in 2003 by Burmester *et al.* ^2^ This was conducted in unsuccessful cases of endoscopic retrograde cholangiopancreatography as well as cases in which reaching the papilla was difficult due to a surgically altered anatomy or duodenal stenosis.^3^ In addition, EUS–HGS has been performed for biliary obstruction in benign diseases.^4^ However, certain procedural factors, such as puncture site, dilation method, and stent selection, still need to be addressed in order to optimize EUS–HGS.

**FIGURE 1:**
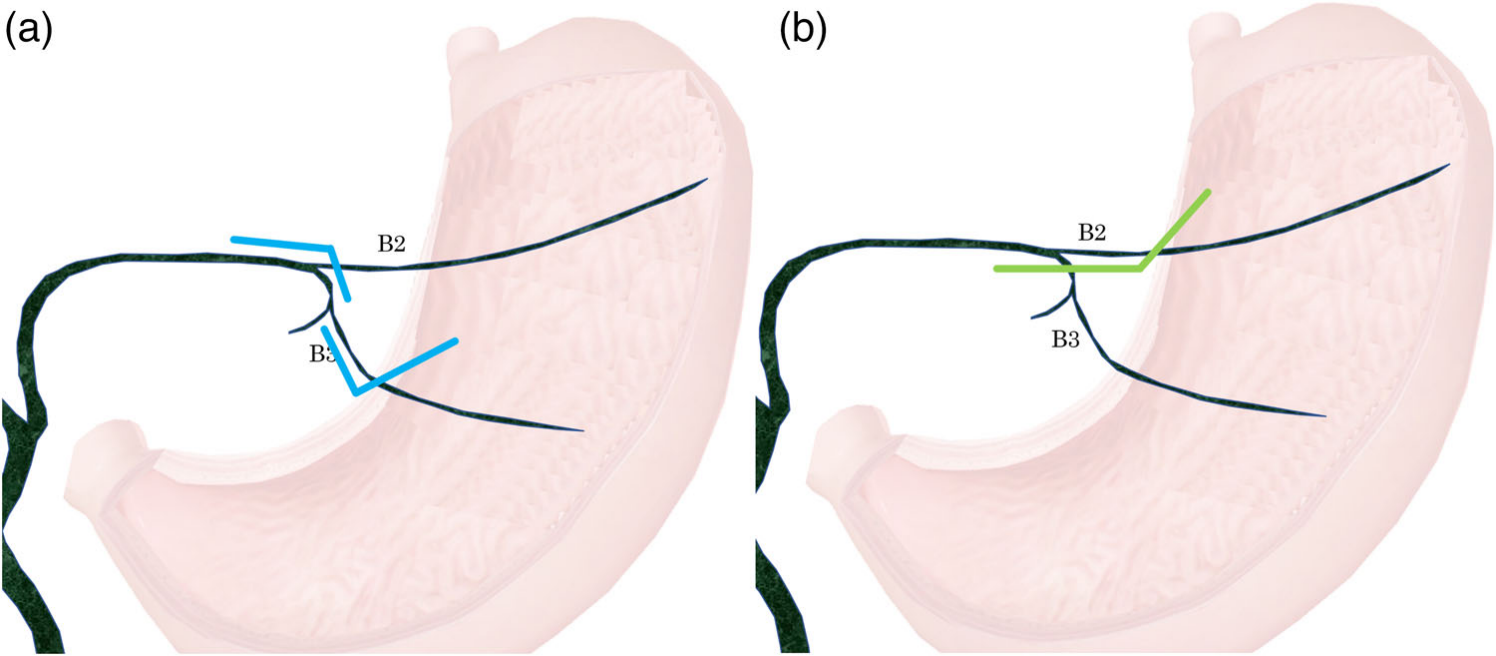
The comparison of guidewire route bending in B2 and B3. (a) B3 puncture includes the following two bends: bending between the puncture needle and the bile duct B3 branch, and bending at the confluence of B2/B3. (b) B2 puncture includes bending at one location, between the puncture needle and the bile duct B2 branch.

The most challenging technique for EUS–BD is the guidewire placement. A national survey in Spain reported that reasons for technical failure in 68.3% (28/41) of EUS‐guided cholangiopancreatography cases were guidewire maneuver related.^5^ In EUS–HGS, especially, puncture via the bile duct of segment 3 (B3; P‐B3) is preferably selected in order to avoid the risk of tracheoesophageal puncture,^6^, ^7^ as puncture via the bile duct of segment 2 (B2; P‐B2) may incur mediastinal injury. After P‐B3, it is sometimes difficult to subsequently maneuver the guidewire into the bile duct. A recent study reported on the puncture angle that facilitated guidewire placement in P‐B3 during EUS–HGS.^8^ In terms of the ease of guidewire placement, P‐B2 is superior to P‐B3 with regard to the puncture angle for the intrahepatic bile duct, bending angle in the common bile duct direction, and the number of bends (Figure 1). However, P‐B2 has a potential risk of puncturing the esophageal wall through the mediastinum and requires special attention be paid in order to prevent mediastinitis and pneumomediastinum.^9^

In this study, we conducted a comparative investigation of P‐B2 and P‐B3 using multicenter retrospective data pertaining to EUS–HGS.

## METHODS

### Patient background

We retrospectively investigated 161 cases of EUS–HGS conducted at the following 6 facilities from April 2015 to July 2021: Jichi Medical University, Saitama Medical Center, National Cancer Center Hospital East, Dokkyo Medical University, Saitama Medical Center, National Hospital Organization Disaster Medical Center, Toyooka Hospital, and Showa University Fujigaoka Hospital.

Indications were determined at the discretion of the endoscopists at each facility. We selected cases in which cannulation failed as well as those with difficulty in reaching the papilla due to gastric outlet obstruction and surgically altered anatomy. We also included cases in which, even if reached, approaching the bile duct from the papilla or jejunal anastomosis was difficult. We conducted the procedure with informed consent for not only malignant but also benign disease cases. The study protocol was approved by the Institutional Review Board of each hospital.

### EUS–HGS procedure

We used a linear echoendoscope (GF‐UCT 240 or GF‐UCT260, Olympus Medical Systems, Tokyo, Japan; EG 580 UT, Fujifilm Corp., Tokyo, Japan) for all cases. A sedative was used, and the components of the sedative were chosen at the discretion of each facility.

First, we visualized B2 and B3 in the left lateral lobe of the liver. The B2 and B3 puncture sites were selected at the discretion of the endoscopists in each facility. The procedure was conducted with a 19 or 22‐G puncture needle; the needle was also selected at the discretion of the endoscopists in each facility. After the puncture, the guidewire was placed in the intrahepatic bile duct, common bile duct, duodenum, or jejunum. We placed the stent either after dilation or without dilation. The performance and type of dilation were selected at the discretion of the endoscopists in each facility. Dilation was accomplished using either a mechanical, balloon, or electrocautery dilator.

Stents were selected at the discretion of the endoscopists at each facility. Devices used were as follows: a plastic stent (Through and Pass Type‐IT 7‐Fr, 14 cm; 8‐Fr, 15 cm; Gadelius Medical, Tokyo, Japan; Flexima 7‐Fr, 7, 10, 12, 15 cm; 8.5‐Fr, 12, 15 cm; Boston Scientific Japan, Tokyo, Japan; QuickPlace V 7‐Fr, 11, 15 cm; 8.5‐Fr, 11, 15 cm; Olympus Medical Systems, Tokyo, Japan), a self‐expanding metallic stent (SEMS; WallFlex fully covered 10 mm × 6 cm, Boston Scientific Japan, Tokyo, Japan; HANAROSTENT fully covered 6 mm × 10 cm, 8 mm × 10 cm, MI Tech, Gyeonggi‐do, Korea; Niti‐S S‐type partially covered 8 mm × 10 cm, 8 mm × 12 cm, 10 mm × 9 cm, fully covered 6 mm × 12 cm, Taewoong Corporation, Seoul, South Korea).

The patients were divided into the P‐B2 and P‐B3 groups, according to the final successful puncture route. There were four cases in each group where the puncture route was switched from B2 to B3 or from B3 to B2. In all of these cases, the procedure was successful after the puncture route was changed; these cases were included in the P‐B3 and P‐B2 groups, respectively. The reasons for switching the puncture route from B2 to B3 were the failure of puncture or guidewire manipulation, whereas the reasons for switching the puncture route from B3 to B2 were the failure of puncture, guidewire manipulation, or stent placement.

The procedure time was defined as the duration from the initial puncture of the bile duct to the placement of the stent. To evaluate the factors related to the procedure time, we divided the patients into the procedure time <40 min and procedure time ≥40 min groups after excluding patients for whom the puncture route was changed and conducted a subgroup analysis. A period of 40 min was selected as it was an approximate average time of all patients.

### Definitions of technical success, functional success, and adverse events

Technical success was defined as the placement of a plastic stent or SEMS in an appropriate position between the intrahepatic bile duct and the stomach. Functional success was defined as a 50% decrease in or normalization of the serum total bilirubin level within 2 weeks according to Tokyo criteria 2014.^10^ Adverse events were defined as any procedure‐related complications occurring within 2 weeks.^11^

### Statistical analyses

We used EZR (version 1.41; Jichi Medical University, Saitama Medical Center, Saitama, Japan)^12^ for statistical analyses. We used the paired *t*‐test for comparisons between two groups. Factors related to the procedure time were analyzed by a multivariate Cox regression analysis. A *p*‐value <0.05 was considered statistically significant.

## RESULTS

Patient characteristics are shown in Table 1. There were 92 P‐B2 cases and 69 P‐B3 cases. According to the baseline demographics, there were no significant differences in gender, age, or performance status between the groups (*p*‐value 0.723, 0.213, 0.935). There were also no significant differences between the groups in the primary disease causing the obstruction and whether the site of obstruction was perihilar or distal (*p*‐value 0.24, 0.144).

**TABLE 1:** Patient characteristics

		B2	B3	*p*‐Value
Gender	Male	60	47	0.723
	Female	32	22	
Age (years, mean, range)		66.9 (32–90)	68.6 (32–87)	0.213
PS	0	25	28	0.935
	1	43	32	
	2	17	8	
	3	6	1	
	4	1	0	
Diagnosis	Pancreatic cancer	44	22	0.240
	Biliary tract cancer	14	28	
	Gastroduodenal cancer	19	8	
	Bile duct stone	3	1	
	Malignant disease	6	3	
	Benign disease	6	7	
Site of obstruction	Distal bile duct	54	35	0.144
	Perihilar bile duct	35	30	
	Anastomosis	1	2	
	Ampulla of Vater	0	1	
	No stenosis	2	1	

The results are shown in Table 2. The technical success rates were 100% (92/92) in P‐B2 and 98.6% (68/69) in P‐B3, with no significant differences between the groups. The functional success rates were 83.7% (77/92) in P‐B2 and 92.8% (64/69) in P‐B3, with no significant differences between the groups.

**TABLE 2:** Treatment results

		B2	B3	*p*‐Value
Technical	Success	92	68	0.321
	Failure	0	1	
Functional	Success	77	64	0.935
	Failure	15	5	
Dilation	None	7	5	0.000285
	Mechanical	55	29	
	Balloon catheter	15	20	
	Electric catheter	15	15	
Stent	Plastic	74	40	0.0776
	SEMS (full covered)	11	14	
	SEMS (partial covered)	7	15	
Procedure time (min, mean, range)		35.2 (8–110)	47.0 (9–187)	0.00601
Adverse event	None	80	55	0.306
	Mediastinal emphysema	1		
	Sepsis	2	2	
	Ulceration		1	
	Bleeding	2	2	
	Peritonitis	2	4	
	Bile leakage	3	1	
	Pancreatitis	1		
	Stent slippage	1	2	
	Stent migration		1	
	Liver abscess		2	

Abbreviation: SEMS, self‐expanding metallic stent.

Regarding fistula dilation, P‐B2 had a significantly higher use of mechanical dilators (59.8% [55/92]) than other dilater (balloon catheter: 16.3% [15/92], electric catheter: 16.3% [15/92]), whereas P‐B3 showed no marked difference in the use of any dilator (mechanical dilator: 42.6% [29/68], balloon catheter: 29.4% [20/68], electric catheter: 22.1% [15/68]). Regarding stent type, P‐B2 had a higher rate of plastic stent use, whereas P‐B3 had a higher rate of SEMS use. Regarding the average procedure time, that for P‐B3 was 47.0 min, and that for P‐B2 was significantly shorter at 35.2 min (*p*‐value, 0.00601). P‐B2 was associated with 12 adverse events (13.0%), whereas P‐B3 was associated with 14 adverse events (some overlapping) (20.1%), with no significant differences noted between the groups (*p*‐value, 0.306). There were no serious adverse events in either group.

We did not measure the procedure time separately for puncture, guidewire manipulation, tract dilation, and stenting. To evaluate the factors related to the procedure time, we divided the patients into the procedure time <40 min and procedure time ≥40 min groups after excluding patients in whom the puncture route was changed (Table 3). In the multivariate analysis, the puncture site—but not the dilation method or stent type—was identified as a factor related to the procedure time.

**TABLE 3:** Multivariate analysis for the procedure time

		Procedure time <40 min (*n* = 89)	Procedure time ≥40 min (*n* = 64)	Multivariate	
				OR (95% CI)	*p*‐Value
Puncture site	B2	61	27	2.83 (1.42–5.64)	0.00315
	B3	28	37		
Dilation	None	2	7	0.928 (0.615–1.40)	0.720
	Mechanical	53	27		
	Balloon catheter	19	15		
	Electric catheter	15	15		
Stent	Plastic	69	38	1.25 (0.764–2.05)	0.373
	SEMS (full covered)	8	17		
	SEMS (partial covered)	12	9		

Abbreviations: CI, confidence interval; OR, odds ratio; SEMS, self‐expanding metallic stent.

## DISCUSSION

In this study, we investigated safety and clinical performance of EUS–HGS, depending on the puncture route of B2 or B3 in the left lateral lobe. We showed that P‐B2 is as effective as P‐B3 with regard to technique and safety. P‐B2 may be a useful puncture route in EUS–HGS.

Endoscopic retrograde cholangiopancreatography is generally the treatment for BD of obstructive jaundice. EUS–BD is selected for cases in which cannulation is difficult or the papilla is difficult to reach due to duodenal obstruction or a surgically altered anatomy. The majority of EUS–BD procedures associated with BD are EUS‐guided choledochoduodenostomy or EUS–HGS. Reports comparing the usefulness of each procedure have indicated that EUS‐guided choledochoduodenostomy had a superior stent patency rate to that of EUS–HGS.^3^, ^13^ EUS–HGS is suited for cases in which approaching from the duodenum is difficult (i.e., those with a surgically altered anatomy). Furthermore, there are some reports of EUS‐guided hepaticojejunostomy, which is a transintestinal approach from the elevated jejunum following total gastrectomy; hence, EUS–HGS has the advantage of broader clinical indications. There is some technical consensus regarding the EUS–HGS procedure^11^, ^14^; however, discussion regarding procedural factors, such as the puncture site, dilation method, and stent selection needed to minimize procedural complications, is still ongoing. In this study, we conducted a comparative investigation of B2 and B3 as potential puncture sites.

P‐B3 is mainly conducted to minimize the risk of tracheoesophageal puncture, which is more likely with P‐B2.^6^, ^7^ However, it is sometimes difficult to subsequently maneuver the guidewire in P‐B3. In terms of the ease of maneuvering the guidewire, P‐B2 seems superior to P‐B3; however, P‐B2 carries a potential risk of esophageal wall puncture. Methods of avoiding tracheoesophageal puncture during P‐B2 include placing a landmark clip at the EG junction, using forward‐viewing EUS,^15^ and checking the shape of the scope, which looks up from the foot side to the head side on fluoroscopic imaging. Mediastinal puncture can be avoided, even when the puncture is via the intraabdominal esophagus caudal to the crus muscle, by carefully recognizing the crus muscle under endosonographic imaging. In addition, newer 0.018‐in. guidewires have enabled the use of 22‐G puncture needles, which facilitates safer puncture than when 19‐G needles are used.^16^, ^17^ When conventional oblique‐viewing EUS is used, tracheoesophageal puncture can be avoided by setting an obtuse puncture angle using a 22‐G needle. Furthermore, the same applies for P‐B3; this allows for an obtuse puncture angle to the intrahepatic bile duct and can reduce the progress of the guidewire to the peripheral intrahepatic bile duct.

**FIGURE 2:**
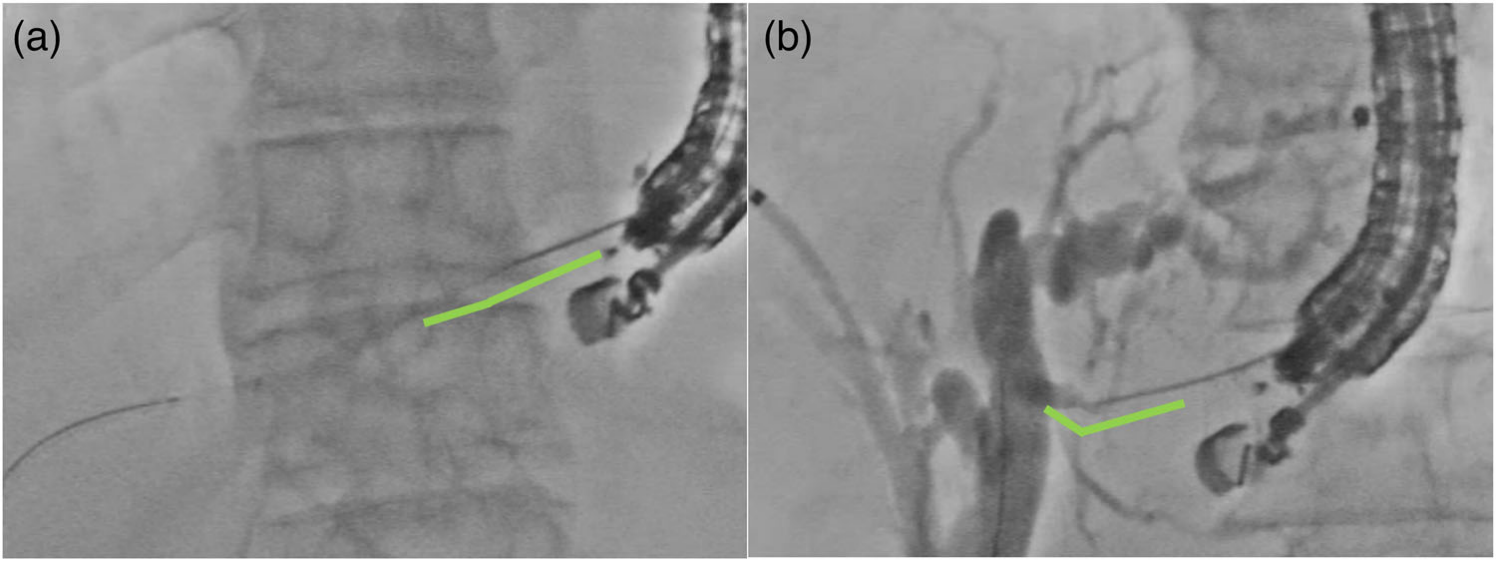
The comparison of the puncture angle for both B2 (a) and B3 (b) in the radiographic image

As with the guidewire placement, fistula dilation and stent placement are simpler with P‐B2 than with P‐B3, because the route to the intrahepatic bile duct is nearly straight (Figure 2). However, even if esophageal puncture is avoided during P‐B2, the mediastinum may be unintentionally breached during the procedure. The mediastinal cavity can be avoided under endosonographic guidance, but the effects on the mediastinum must be considered during fistula dilation and stent placement. Many different mechanical dilators were used for P‐B2 in the present study (Table 2). There are reportedly few adverse events when using mechanical dilators.^18^ Therefore, minimum dilation may be conducted in P‐B2 in order to reduce the effect on the mediastinum thanks to the easy stent delivery in the straight direction. Regarding the stent placement, the number of cases using plastic stents may have increased in consideration of the adverse events caused by stent dilation. In fact, 7‐Fr plastic stents were used in 83.6% (46/55) of P‐B2 cases. In this study, there was only one case of mediastinal emphysema as an adverse event in the P‐B2. In that case, an electrocautery dilator and a fully covered SEMS were used. An electrocautery dilator causes a burn effect on its surroundings.^19^ It is possible that there was an unexpected dilation associated with the electrocautery dilator in this case. In recent years, small‐diameter SEMS with an outer stent diameter of 6 mm and stent delivery of 5.9 Fr have been developed, and it is expected that adverse events, such as bile leakage, could be reduced with little to no dilation.^20^

In the present study, there were no significant differences in the rates of technical success, functional success, or adverse events; however, the total procedure time was shorter in the P‐B2 than in the P‐B3 (Table 2). We should have evaluated the procedure time by separating in puncture, guidewire manipulation, tract dilation, and stenting. However, these data were not available due to the retrospective nature of the present study. To compensate for this, we performed a subgroup analysis in which the patients were divided into the procedure time <40 min and procedure time ≥40 min groups. In the multivariate analysis, the puncture site was the only factor related to the procedure time; the dilation method and stent type were not related to the procedure time. The shorter procedure time in P‐B2 might be due to the reduction in time needed for maneuvering the guidewire during advancement to the proximal bile duct, suggesting the possibility that it may be easier to approach via B2.

Our findings showed that P‐B2 was as useful as P‐B3 and that P‐B2 may be an effective option in EUS–HGS. The usefulness of 0.018‐in. guidewires has also been reported,^21^ and prospective studies on the usefulness of B2 or B3 segment punctures may be conducted in the future. With the use of appropriate device accessories, P‐B2 can safely be considered the first choice in performing EUS–HGS.

Several limitations associated with the present study warrant mention. First, this was a retrospective study. Second, the puncture needle sizes and guidewire sizes could not be compared. There were some lacks in the previous data, and we were unable to investigate the puncture needle sizes in detail. The data of the diameter of the punctured bile duct was also unavailable. However, it is important that this study, which included many cases, demonstrated the usefulness of P‐B2. Third, recurrent biliary obstruction was not evaluated, and the times to recurrent biliary obstruction were not compared. Therefore, the further investigation of long‐term stent patency and re‐intervention is needed. Fourth, we divided the groups according to the successful drainage route. In the aim of the study, this may have a disadvantage about the procedure time, and we may have had to divide the groups according to the initial drainage route. However, we did not measure the procedure time by separating in puncture, guidewire manipulation, tract dilation, stenting, and failure steps. Therefore, we grouped the patients according to the successful drainage route in which most of the procedure time was spent.

## CONCLUSIONS

Puncture via B2 in EUS–HGS may facilitate guidewire placement and manipulation and enable a shorter procedural time. In addition, the P‐B2 procedures were not inferior to those of P‐B3 in terms of the success rate and adverse event rate. An approach via B2 may therefore be considered a useful option for EUS–HGS with appropriate accessory use.

## CONFLICTS OF INTEREST

None.
